# The Cellular and Molecular Signature of ALS in Muscle

**DOI:** 10.3390/jpm12111868

**Published:** 2022-11-08

**Authors:** Ekene Anakor, William John Duddy, Stephanie Duguez

**Affiliations:** Northern Ireland Center for Personalised Medicine, School of Medicine, Ulster University, Derry-Londonderry BT47 6SB, UK

**Keywords:** motor neurone disease, muscle metabolism, neuromuscular junction, muscle regeneration, muscle secretome, muscle-nerve communication

## Abstract

Amyotrophic lateral sclerosis is a disease affecting upper and lower motor neurons. Although motor neuron death is the core event of ALS pathology, it is increasingly recognized that other tissues and cell types are affected in the disease, making potentially major contributions to the occurrence and progression of pathology. We review here the known cellular and molecular characteristics of muscle tissue affected by ALS. Evidence of toxicity in skeletal muscle tissue is considered, including metabolic dysfunctions, impaired proteostasis, and deficits in muscle regeneration and RNA metabolism. The role of muscle as a secretory organ, and effects on the skeletal muscle secretome are also covered, including the increase in secretion of toxic factors or decrease in essential factors that have consequences for neuronal function and survival.

## 1. Introduction

The term Motor Neuron Disease (MND) encompasses several phenotypes, with Amyotrophic lateral sclerosis (ALS) being the most common [1,2]. ALS is a heterogenous, late-onset and rapidly progressive disease characterized by the gradual loss and degeneration of upper and lower motor neurons and with a life expectancy of 2–5 years following diagnosis [1,3]. Patients can either present with lower motor neuron symptoms: denervation, muscle atrophy, fasciculations and cramping or with upper motor neuron symptoms characterized by spasticity, poor coordination, hypotonia and hyperreflexia [4,5,6]. The incidence of ALS is 1–2 persons per 100,000 per year and a prevalence of 5.2 per 100,000, which is expected to rise with aging of the world population [7,8]. The total number of ALS cases for 2020 across 22 countries was estimated at prevalence of 121,028 and incidence of 41,128 [9].

ALS can be familial (fALS), defined by its presence in more than one family member and accounting for 10–15% of known cases, or sporadic (sALS), with a spontaneous onset and accounting for 85–90% cases [10]. The clinical manifestations of either form of the disease are indistinguishable, suggesting a convergence of common pathways or factors that cause a cascade of neurodegeneration [11,12]. There is a genetic component to ALS as mutations in a number of genes (>30) with moderate to high-penetrance are described as monogenic causes of the disease, explaining 15% of sporadic and 66% of familial cases, respectively [10,11,13]. The most commonly studied genes with causal links to ALS include: Superoxide dismutase 1 (*SOD1*; ~20% of fALS and 1–4% sALS), Fused in sarcoma (*FUS*; ~1–5% of fALS and ~1% sALS cases),TAR DNA binding protein 43 (*TARDBP*; ~4–5% of fALS and 2% sALS cases) and chromosome 9 open reading frame 72 (*C9orf72*; 1 in 10 of ALS cases among European-ancestry populations) [14,15]. These genetic variants affect biological processes such as mitochondrial and endoplasmic reticulum (ER) dysfunction, oxidative stress, dysregulated RNA metabolism and impaired axonal transport [16]. Other factors including the environment and ageing are also implicated in the pathology of ALS [17,18].

Increasingly, ALS is considered as a multisystemic disease owing to the involvement of neuronal and glial cells as well as the immune response and skeletal muscle in the disease process [19,20,21,22]. Changes in the cellular structure, physiology and metabolism of the aforementioned cell types appear to occur in a synergistic and mutual way that makes it difficult to pinpoint the primary pathogenic mechanism or cell type responsible for the disease [19]. Skeletal muscle pathology and functional abnormalities occur in fALS, sALS and in ALS murine models [23,24,25,26,27,28], but often more work is needed to fully explore these changes, and the muscle has been highlighted as an understudied tissue in ALS [29].

An interdependent and indispensable relationship exists between the skeletal muscle and motor neurons (MN) during development, with MN dying if deprived from their target muscle and vice versa [30,31,32,33]. The neuromuscular junction (NMJ) represents a crucial site allowing bidirectional communication between the MN, muscle fibers, and terminal Schwann cells [34,35]. MN activity regulates muscle function while the muscle generates retrograde signals that preserve NMJ integrity and function [36]. Different types of MN (alpha, gamma and delta) innervate muscle fibers within skeletal muscle with important functions for motor control [37]. MN are known to drive the development and maintenance (as well as degeneration or dismantling) of NMJs, with skeletal muscle considered as passive in the developmental process of the NMJ as well as during the denervation and dismantling observed in ALS, although this latter view has been challenged [35,38,39]. Cellular elements within the NMJ, such as the MuSK LPRE-Agrin interaction, orchestrate muscle-derived retrograde signaling that is important for NMJ stability and maintenance, and these retrograde elements may potentially be disturbed in ALS [40]. As discussed in Section 4 below, FUS, a key protein associated with ALS, also seems to have a key role in the maintenance of the NMJ [41], as well as MG53, a muscle protein involved in membrane repair [42]. Restricted expression of human SOD1 variants (wild-type-, G37R- and G93A-hSOD1) in the skeletal muscle of mice recapitulated an ALS-type phenotype including age related neurological symptoms, skeletal muscle pathology, including limb weakness, muscle atrophy, myofiber cell death and NMJ abnormalities, as well as MN neuronopathy/distal axonopathy [43,44].

Despite its role in locomotion, skeletal muscle is increasingly considered an endocrine/secretory organ due to its constitutive release of proteins (cytokines and growth factors), myokines, or extracellular vesicles, acting locally on muscle and other cell types via autocrine/paracrine loops [45,46], such as to maintain the pre-synapse and the NMJ [47], or entering the bloodstream to have systemic effects [48]. In ALS, muscle secretion of semaphorin-3A [49] and Nogo-A [50,51], as well as the release of muscle vesicles [21], have all been shown to have a toxic effect on motor neurons.

This review attempts to summarize the different dysfunctions observed in ALS muscles.

## 2. Metabolic Dysfunction in ALS Skeletal Muscle

Defects in mitochondrial structure and function as well as energy metabolism in skeletal muscle contribute to disease pathology and progression, with metabolic dysfunctions appearing long before motor neuron degeneration and death [52].

### 2.1. Mitochondrial Dysfunction

Mitochondria are involved in cellular production of radicals that are implicated in neurodegenerative diseases, and evidence exists for mitochondrial involvement in the skeletal muscle pathogenesis of ALS, exemplified by abnormal mitochondrial aggregates and vacuolations within skeletal muscle in ALS mouse models and human patients/biopsies [53,54] and reviewed by [55]. Furthermore, the excessive presence and accumulation of Reactive Oxygen Species (ROS) as well as ultrastructural mitochondrial abnormalities in the skeletal muscles of mice bearing SOD1^G93A^ mutations and in ALS patients, suggests a disease dependent change in mitochondrial function [24,56]. Excess ROS production in skeletal muscle results in damage to mitochondrial structure and function including mitochondrial DNA deletions and depletions, and decreased activity of respiratory chain mitochondrial enzymes that ultimately leads to decreased generation of ATP [52,57,58]. Interestingly, mitochondrial alterations including a reduction in NADH:CoQ oxidoreductase activity are present in skeletal muscle biopsies obtained from sALS patients, and the severity of mitochondrial dysfunction increased as ALS worsened [24,58,59,60], suggesting that dysfunctional mitochondria are a common trait in ALS skeletal muscles.

### 2.2. Dysregulated Skeletal Muscle Energy Metabolism (Hypermetabolism and Dyslipidemia)

Closely linked to mitochondrial dysfunctions, energy dyshomeostasis represents an early and persistent observation during ALS [61]. Abnormalities in skeletal muscle energy metabolism provokes a defect in energy homeostasis exhibited as decreased glycolysis, elevated beta-oxidation in skeletal muscles as well as hypermetabolism [62,63], and may be responsible for the NMJ dismantling seen in a transgenic mouse model (SOD1^G86R^/MCK-UCP1 mice) [64,65]. An ALS-associated protein, TDP-43 regulates body fat composition and skeletal muscle glucose homeostasis in vivo—transgenic mice overexpressing wild type TDP-43 possess myofibers with altered Glut4 translocation and glucose uptake abnormalities [66]. Muscle specific expression of SOD1^G93A^ in MLC/SOD1^G93A^ mice induces fatty acid (FA) recruitment to skeletal muscle that is necessary for sustaining lipid flux into skeletal muscle fibers and ensuring availability of FAs for β-oxidation—these likely precede muscle denervation and have also been identified in ALS patients and other murine models [52,67]. In addition, hypermetabolism, defined as an abnormally elevated level of resting energy expenditure, has been identified in sALS (25–68% prevalence) and fALS (100% prevalence) patients, with its presence in familial cohorts suggesting a genetic component even though the exact cause(s) remain unknown and is associated with poor prognosis [68]. Clinically, weight loss is a symptom of ALS and body mass index (BMI) has been inversely correlated with ALS risk and progression, with lower BMI values in ALS cohorts associated with malnutrition arising from dysphagia [52].

Overall, mitochondrial dysfunction and energy deficits or hypermetabolism in ALS skeletal muscles are linked, having a capacity to cause NMJ disruption and, consequently, motor neurodegeneration [69,70,71].

## 3. Impaired Muscle Proteostasis

Protein homeostasis is a finely regulated process that is a balance between protein production/synthesis, folding and degradation involving protein quality control, trafficking and clearance, and it is important for preventing cellular dysfunction and propagation of misfolded proteins [72]. Skeletal muscle cells handle protein misfolding more efficiently than MNs, clearing misfolded proteins, including mutant SOD1 and aggregate species of TDP-43, using a combination of an efficient proteasome and autophagic system [73,74]. Protein quality control system includes molecular chaperones and proteolytic mechanisms such autophagy, ubiquitin-proteasome and the unfolded protein response pathways are more efficiently activated in skeletal muscles than the nervous system and appear more relevant in the skeletal muscle [75,76,77], with a significant suppression of autophagic flux in skeletal muscles of SOD1^G93A^ ALS model as the disease progresses [78]. Indeed, TDP-25 (prion-like TDP-43 fragment) mislocalizes to the cytoplasm forming aggregates in myoblasts (although to a lower extent when compared to MNs) and impairs autophagy [74], and pTDP43 inclusions were observed in ALS patient muscles [74,79] especially in axial skeletal muscles [79,80]. Furthermore, proteasome activity in the skeletal muscle of SOD1^G93A^ mice is upregulated in early symptomatic stage with a reduction as the disease progresses towards symptomatic and terminal stages whereas autophagic activation in the skeletal muscle of SOD1^G93A^ mice occurs at presymptomatic and terminal stages [81]. Impairment in proteostasis may be due in part to mutations in ALS-associated proteins including SOD1, p62, valosin-containing protein (VCP), ubiquilin-2 (UBQLN2), optineurin (OPTN), and TANK-binding kinase 1 (TBK1) [82,83].

## 4. Dysregulated RNA Metabolism in Skeletal Muscle

RNA-binding proteins such as TDP-43 (transactivation response element DNA-binding protein 43), FUS, TAF15 (TATA-binding protein-associated factor 15), EWSR1 (Ewing sarcoma breakpoint region 1), and heterogeneous nuclear ribo-nucleoproteins A1 and A2 (hnRNPA1 and hnRNPA2) are involved in aspects of RNA metabolism including mRNA transcription and stabilization, alternative splicing, RNA transport, and miRNA biogenesis [16,84,85]. They possess RNA recognition motifs and low complexity or prion-like domains that could act as key scaffolds, causing them to accumulate in cytoplasmic stress granules or other membrane-less organelles such as nuclear paraspeckles implicated in ALS [86].

TDP-43 is essential for myoblast differentiation and skeletal muscle regeneration in vitro and in vivo studies, respectively [87]. Cytoplasmic TDP-43 is involved in the formation of temporary structures or amyloid-like assemblies called myo-granules that contain mRNAs encoding for sarcomere associated proteins during normal skeletal muscle formation, and which are increased during regeneration in response to damaged skeletal tissue [87]. Over time, myo-granules from C_2_C_12_ myotubes seeded amyloid-like fibrils in vitro, revealing a mechanism by which TDP-43 aggregation could occur [87]. In addition, phosphorylated TDP-43 (pTDP-43) aggregates were observed in the skeletal muscles of sALS and fALS cohorts, highlighting axial skeletal muscles as an additional site of phosphorylated TDP-43 pathology, while suggesting that impaired proteostatic clearance of misfolded proteins might play a role in the pathology [79]. On the other hand, FUS which is involved in aspects of RNA processing and metabolism, was significantly enriched in subsynaptic myonuclei but this enrichment was lost upon denervation and disrupted in the presence of mutant ALS FUS, highlighting mutant FUS toxicity to skeletal muscle in vivo [41]. Furthermore, muscle biopsies from patients with *FUS* mutations revealed muscle atrophy and a loss in FUS subsynaptic enrichment [41]. In addition, mutant FUS toxicity to skeletal muscle was demonstrated in MN-myotube co-cultures and observed toxicity was due to endplate maturation defects arising from inefficient gene expression of acetylcholine receptor subunits in subsynaptic myonuclei as well as impaired myogenic differentiation **[41]**. Finally, products of the *C9orf72* hexanucleotide repeat expansion (HRE), G4C2 RNA foci and dipeptide repeat (DPR) proteins have been identified in skeletal muscles of animal models of C9-ALS [88,89] and in the skeletal muscles of ALS patients with *C9orf72* mutations [90,91]. Although Swartz and colleagues reported lower levels of *C9orf72* variants in iPSC skeletal muscles compared to iPSC-derived MNs and an absence of TDP-43 mislocalization or ubiquitin/p62-positive inclusions [91], another study showed that C9-ALS myocytes from iPSCs of *C9orf72* ALS patients had increased expression and aggregation of TDP-43 [92], necessitating the need for more studies to investigate contribution of *C9orf72* transcripts to skeletal muscle pathology.

Epigenetic regulation, especially that mediated by microRNAS (miRNAs) and histone deacetylases or HDACs, are affected in ALS skeletal muscle [93]. At neuromuscular synapses, miR-206 and HDAC4 control the denervation-innervation process and both are proposed to be involved in ALS progression as their expression levels are altered in the skeletal muscles of animal models and ALS patients [94,95,96,97]. Upregulation of HDAC4 mRNA in muscle biopsies of ALS patients correlates with disease severity, with its expression being higher in patients with faster disease progression [96]. Furthermore, HDAC links neuronal activity and muscle transcription during denervation since HDAC4 is normally concentrated at the neuromuscular junction with low levels in skeletal muscles [98]. HDAC4 may have a role in muscle pathology, with its expression increasing during skeletal muscle denervation, activating the muscle atrophy [98]. On the other hand, miR-206 is a negative regulator of skeletal muscle HDAC4 [99] and may have a role in NMJ maintenance [100]. In muscle biopsies from patients with genetic forms of ALS (*SOD1* and *C9orf72* mutations), miR-206 level is strongly increased while the HDAC4 protein expression is decreased [95]. This elevated expression of miR-206 at the onset of neurological symptoms is suggested to be a compensatory mechanism aimed at re-establishing the connection between the muscle and nerve via reinnervation [95].

## 5. Defects in Muscle Regeneration and Resident Stem (Satellite) Cell Behaviour

ALS is associated with an impairment of skeletal muscle satellite cell (SC) activity and myogenic potential [22], these being required to drive myogenesis in response to cues such as acute injury or denervation [101]. Upon activation, SCs leave their quiescent state, entering into proliferation, fusion and differentiation [102,103]. Muscle cells extracted from ALS patient muscle biopsies, although not presenting a reduction in their proliferation capacity [104], did present an impaired capacity to differentiate into myotubes when compared to healthy muscles [105,106,107]. Interestingly, in a recent study of the C9rof72 murine model with 36 × G4C2 repeats [108], fibers with chains of central nuclei could be observed—a feature typical of degenerative muscle as observed in the mdx mouse [109]. Post-transcriptionally, muscle specific miRNAs (or myomiRs—a subset of miRNAs specific to striated muscle, including miR-206) are upregulated and preserve NMJ after nerve injury by allowing for reinnervation [94]. Skeletal muscle biopsies of ALS patients carrying *C9orf72* and *SOD1* gene mutations, as well as SOD1 mice, displayed elevated levels of myomiRs including miR-206 that coincided with the onset of neurological symptoms and especially at the symptomatic stage [110,111].

The cross-talk between the immune system and satellite cells is necessary for muscle regeneration [112,113], representing another way in which inflammation could play a role in ALS skeletal muscle pathology [114]. Macrophage infiltration has been reported in fibrotic tissue in patient muscles [115] and in ALS rat models [115,116,117] with elevated levels of inflammatory cytokines have been reported in the skeletal muscles of ALS rat models [116], while degranulating mast cells and neutrophil accumulation contribute to denervation and are present in myofibers and motor endplates derived from ALS patients and SOD1^G93A^ rats [115,117].

## 6. The Skeletal Muscle Secretome in ALS

Profiles of the skeletal muscle secretome in animal and human models include several hundreds of cytokines, proteins and peptides, lipids, amino acids, metabolites, and small RNAses, as well as extracellular vesicles (small EVs and ectosomes), suggesting an important role of the secretome in intercellular communication [118,119].

### 6.1. Factors Involved in Neuromuscular Junction Maintenance

Skeletal muscle secreted factors including β-catenin or β1 integrin continuously modulate development and maintenance of the NMJ and are dysregulated in response to pathological changes [120,121]. The retrograde signaling is important for NMJ development and integrity as it is the process by which skeletal muscle regulates and maintains normal functioning of the components of the NMJ [122]. For example, muscle fibers regulate presynaptic differentiation and function via muscle secreted β-catenin or β1 integrin acting in a retrograde manner [121]. Skeletal muscle-secreted C1q/TNF-related protein 3 (CTRP3) contributes to neuronal physiology by regulating neuronal protein synthesis, axonal outgrowth and protein synthesis rate in an mTOR dependent manner, and CTRP3 levels were reduced in the muscle secretome, serum and muscle tissues of a spinal muscular atrophy (SMA) animal model [123].

Skeletal muscle toxicity may contribute to retrograde degeneration signaling cascade, elicited as an increase in the secretion of toxic and destabilizing factors or a decrease in essential signaling molecules, that causes axon retraction, axonopathy and consequently, motor neuron degeneration [124]. Microfluidic co-cultures of myocytes with *SOD1^G93A^*, *TARDP* or *C9orf72* mutations and mouse embryo spinal cord explants revealed the inability of axons to cross to the distal compartment (housing myocytes) and either retracted, remained static or underwent degeneration possibly in response to dysregulated secretion of factors from myocytes [125].

In response to ALS mutations, toxic and destabilizing factors can be secreted from skeletal muscles that contribute to NMJ disruptions. Semaphorin3A (SEMA3A) is an axonal guidance protein that acts as an axon repellent, preventing axonal regeneration by binding to its co-receptor, neuropilin1 (NRP1), and increased production of the protein is observed in the skeletal muscles of ALS animal models [125,126], NMJs of fast-fatigable muscle fibers and terminal Schwann cells [127], and the motor cortex of ALS patients [128]. Moreover, ALS mice with specific knockout of the Semaphorin3A receptor, NRP1, or treated with a monoclonal antibody interfering with SEMA3A-NRP1 signaling, exhibited improved function and prolonged survival [129,130]. Semaphorin3A is also suggested to be involved in formation of skeletal muscle retrograde toxic signaling, facilitated by Collapsin Response Mediator Protein 4 (CRMP4)-dynein interaction, and contributing to MN loss [131]. Like semaphorin3A, Nogo-A is a neurite outgrowth inhibitor implicated in: growth cone collapse, restriction of axonal regeneration, and repair of injured axons [132]; as well as synapse integrity and stabilization [133]. Skeletal muscles of SOD1^G93A^ mice and sporadic ALS patients showed ectopic levels of Nogo-A [134,135] that correlated with disease progression [136].

Muscle fibers express and concentrate fibroblast growth factor binding protein 1 (FGFBP1) at the NMJ, with FGFBP1 implicated in promoting maturation and repair of the NMJ [94]. In SOD1^G93A^ mice, the loss of FGFBP1 accelerated muscle denervation and progression of ALS [120]. Other examples of muscle secreted factors important for NMJ stabilization and dysregulated in ALS animal models and patients include: Insulin Like Growth Factor-1 (IGF-1) [137,138,139,140] or Glial-Cell-Line-Derived Neurotrophic Factor (GDNF) [141,142,143,144,145], Irisin [146] and Brain-Derived Neurotrophic Factor (BDNF) [138,147].

Post-transcriptional regulators such as microRNAs (miRs) can also contribute to the disruption of the NMJ as they (and their target genes) are present in the skeletal muscle secretome [148]. MyomiRs are effective regulators of muscle homeostasis, plasticity and myogenesis in physiological and pathological conditions [149]. They are important for the development and maturation of neuromuscular synapses, and their dysregulation has been linked to ALS associated degeneration [150,151]. miR-206 is upregulated in patients and animal models of ALS and is correlated with disease onset (symptomatic phase), likely as a compensatory mechanism, while miR-133b and miR-1 are dysregulated in ALS skeletal muscle, spinal cord and patient muscle biopsies, respectively, [105,152]. Downregulation of miR126-5p in the skeletal muscles of male ALS mouse models contributes to ALS pathology by facilitating axonal degeneration and NMJ disruption possibly via increased expression and secretion of their targets: axon destabilizing Type 3 Semaphorins and their co-receptors, Neuropilins [125,153]. More importantly, miR126-5p overexpression in in vitro (SOD1^G93A^ myocyte cultures) and in vivo models (*SOD1^G93A^* mice) was sufficient to transiently rescue axon degeneration, NMJ disruption and inhibit the neurodegenerative process [125].

### 6.2. Skeletal Muscle-Derived Extracellular Vesicles: What Is Known

Extracellular vesicles (EVs), especially small extracellular vesicles or exosomes, have been investigated in the pathogenesis of neurodegenerative diseases including ALS [21,154]. Small EVs possess physiological and pathological functions; are important mediators in intercellular and long-distance communication as they can cross the blood–brain barrier and blood-cerebrospinal fluid-barrier, gaining access to the CNS [155] and possibly propagate the spread of pathogenic proteins in neurodegenerative diseases [154,156,157]. While small EVs secreted at the presynaptic terminals have been described to be taken up by the skeletal muscle at the NMJ and have an impact on muscle function [158,159,160], EVs secreted by the muscle can also have an impact on MN [21,161,162]. This section will focus on the role of skeletal muscle derived small EVs (MuVs) in ALS.

MuVs are implicated in muscle-muscle communication involving myoblasts and myotubes with documented roles in myogenesis, muscle regeneration and myoblast differentiation [104,163,164,165,166,167]. MuVs are involved in bidirectional muscle-neuron communication that is responsible for synaptic growth and plasticity, increasing neuronal cell survival, neurite outgrowth and neuron regeneration accuracy [161,168,169]. Generally, in vivo cell-to-cell transfer of administered small EVs is suggested to occur by retrograde axoplasmic transport involving nerve terminals, or movement through interstitial spaces and hematogenous transport via systemic circulation to reach the intended target or recipient cells [170,171,172]. Interestingly, masseter muscle-derived MuVs in response to contraction can be transported along the trigeminal nerve to the hippocampus in a retrograde manner [170]. Similarly, when CD63 enriched small EVs secreted by bone marrow mesenchymal stem cells were injected into gastrocnemius muscle, these small EVs were transferred to motor neurons and glial cells via a combination of retrograde movement along nerve terminals, and released into the bloodstream, with hematogenous transport requiring time to reach recipient cells [173].

MuVs encapsulate myomiRs such as miR-1, miR-133a, miR-133b, and miR-206 that are important for local communication and function between muscle tissues, and have been detected in the blood stream [174]. Furthermore, the myomirs miR-1, miR-133a, and miR-206 are selectively sorted into and elevated within small EVs obtained from the serum of patients with muscular dystrophies—elevation suggested to occur in response to muscle degeneration-regeneration cycle [175]. MuVs derived from denervated myofibers show elevated levels of the miR-206-myomiR also known to be upregulated during myoblast differentiation and muscle regeneration [176]. This marked increase would suggest that denervation provokes the release of myomiR enriched MuVs that assist with local muscle repair or be released into systemic circulation.

MuVs oversecretion was observed in muscles obtained from biopsies of sporadic ALS patients, some with C9orf72 or ATXN2 mutations [21,162]. The MuVs affected RNA processing in recipient motor neurons, causing motor neuron toxicity exhibited as shortened neurites, less branching and motor neuron death [21]. Interestingly, the toxicity associated with skeletal muscle derived exosomes are specific for that vesicle type as ectosomes isolated from myotubes of ALS patients had no effect on motor neurons, and did not affect neurite length or branching or result in motor neuron death [162].

Other studies highlighted that toxic factors secreted by the MuVs include: semaphorin-3A [49]; and Nogo-A [50,51]. Together, these studies suggest a potential role of MuVs in the propagation of neurotoxic elements.

Although initially considered to be only a means for cellular garbage disposal, EVs are now considered to be a common vehicle for intercellular communication. They could shuttle misfolded proteins out of the cell in order to compensate for impaired proteostasis or cellular auto-toxicity as observed in sporadic and familial ALS [16]. In this sense, they could be considered a protective mechanism, the cell’s attempt to correct for internal toxicity. However, EVs can act locally and distantly, being taken up by different cell types, including neuronal and glial cells [177]. In this way, they could spread or propagate toxicity.

## 7. Conclusions

**Figure 1:**
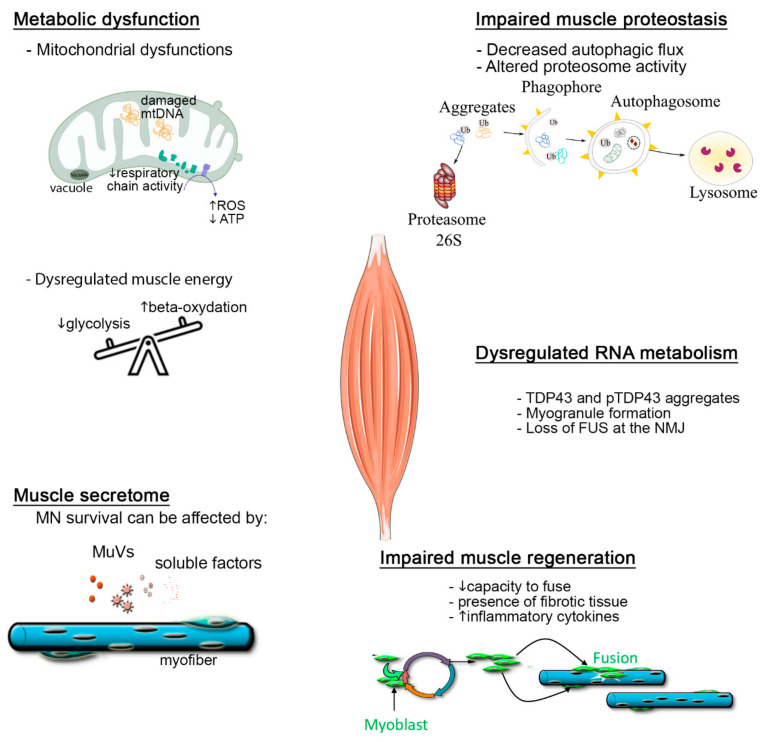
Summary of the cellular and molecular events affected in the skeletal muscles of ALS patients and models.

Numerous cellular and molecular events are affected in the skeletal muscles of ALS patients and models (Figure 1). These include dysfunctions of cell metabolism, of the removal and degradation of proteins and their aggregates, of RNA processes, and of muscle atrophy pathways. Muscle stem cells of patients are affected, presenting a lower capacity for differentiation. Lastly, alterations to the muscle secretome occur, which can be associated with neuronal pathology, either by a loss of neurotrophic factors or by a gain of neurotoxicity, some or all of this neurotoxicity being mediated by muscle-secreted extracellular vesicles, so that the muscle secretome has a potential role in inter-cellular propagation of the disease.

## Data Availability

Not applicable.
